# Replacing school and out-of-school sedentary behaviors with physical activity and its associations with adiposity in children and adolescents: a compositional isotemporal substitution analysis

**DOI:** 10.1186/s12199-021-00932-6

**Published:** 2021-01-27

**Authors:** Aleš Gába, Jan Dygrýn, Nikola Štefelová, Lukáš Rubín, Karel Hron, Lukáš Jakubec

**Affiliations:** Faculty of Physical Culture, Palacký University Olomouc, třída Míru 117, 779 00 Olomouc, Czech Republic

**Keywords:** Accelerometry, Compositional data analysis, Schools, Time-use epidemiology

## Abstract

**Background:**

Little is known on how context-specific sedentary behaviors (SB) affect adiposity. This study aimed to investigate compositional associations between context-specific SB and adiposity and estimate the differences in adiposity associated with replacing school and out-of-school SB with physical activity (PA).

**Methods:**

This study included 336 children and adolescents. Time spent in SB and PA was estimated using multi-day 24-hour raw accelerometer data. SB and PA were specified for school and out-of-school times. Fat mass percentage (FM%) and fat mass index (FMI) were used as adiposity indicators. A compositional isotemporal substitution model was used to estimate differences in adiposity associated with one-to-one reallocations of time from context-specific SB to PA.

**Results:**

Participants spent approximately two thirds of their school and out-of-school time being sedentary. Relative to the remaining 24-h movement behaviors, significant associations between out-of-school SB and adiposity were found in both boys (*β*_ilr1_ = 0.63, 95% confidence interval [CI] = 0.03–1.22 for FM%; *β*_ilr1_ = 0.76, 95% CI = 0.03–1.49 for FMI) and girls (*β*_ilr1_ = 0.62, 95% CI = 0.25–0.98 for FM%; *β*_ilr1_ = 0.80, 95% CI = 0.28–1.32 for FMI). Replacing 30 min/day of out-of-school SB with out-of-school light PA decreased FM% by 10.1% (95% CI = 3.3–17.9) and FMI by 14% (95% CI = 2.7–24) in girls. No significant associations were found for school SB.

**Conclusions:**

A reduction of out-of-school SB in favor of light PA should be advocated as an appropriate target for interventions and strategies to prevent childhood obesity.

**Supplementary Information:**

The online version contains supplementary material available at 10.1186/s12199-021-00932-6.

## Introduction

Sedentary behavior (SB) is described as any waking behavior where energy expenditure is equal to or less than 1.5 metabolic equivalents while a person is sitting, reclining, or lying [1]. SB is considered a predominant part of the 24-h cycle [2] with a high potential to affect health [3, 4]. Excessive SB is highly prevalent among school-aged children and adolescents [5]. The large amount of time spent in SB leads to a dysregulation in the energy balance, which is one of the key contributors to childhood obesity [6].

Numerous studies have investigated the relationship between total SB and adiposity in past decades but their conclusions have been inconsistent [7]. The occurrence of a few unexpected findings [8, 9] suggests a need for an extended investigation. The context in which SB occurs may contribute to an explanation of this inconsistency [10]. Children and adolescents spend a large proportion of their waking hours at school and most of their school time corresponds to SB [11–14]. However, the amount of time they spend in SB during school time might not be the main driver of childhood obesity because it is highly organized with a lack of opportunity to exercise free choice in movement behaviors. In contrast, out-of-school time corresponds more with the attitudes of children and adolescents and their family background [15] and could be the potential source of obesogenic movement behaviors.

Although previously published studies have shown that both school and out-of-school SB are independently associated with adiposity indicators [11, 16, 17], these studies examined SB in isolation without including the remaining movement behaviors within the 24-h cycle (i.e., physical activity [PA] and sleep). It is known that all movement behaviors interact with each other [2, 18], and therefore, the change in adiposity status associated with a change in SB is partly due to a compensatory change in one or all remaining movement behaviors [19]. For this reason, the relationship between SB and adiposity should be examined in accordance with a 24-h time-use paradigm that integrates all daily movement behaviors [2]. This, however, requires the use of appropriate statistical methods such as compositional data analysis (CoDA), which allows one to analyze associations between SB and adiposity relative to other movement behaviors [19, 20]. Moreover, the employment of isotemporal substitution modeling [21] provides an opportunity to design more effective intervention strategies that focus on reducing sedentary time by estimating the theoretical difference in adiposity resulting from replacing SB with one or all remaining movement behaviors within the day.

To our knowledge, a study based on 24-h movement behaviors to examine the associations between context-specific SB and adiposity data has not been published. The present study sought to fill this gap by (1) investigating compositional associations between context-specific SB and adiposity and (2) estimating the differences in adiposity associated with replacing school and out-of-school SB with PA in the 24-h movement behavior composition.

## Methods

### Participants

This observational cross-sectional study included children and adolescents aged 8 to 18 years who were recruited from urban and rural schools across the Czech Republic. The study’s sampling and design details have been reported elsewhere [18, 22]. Briefly, seven primary and four secondary schools in which students are engaged in the mandated amount of physical education lessons (i.e., 90 min a week) participated in this study. The main inclusion criteria were participant age and good health condition. A total of 907 children agreed to participate in the study on a voluntary basis. Of these, 45 participants were excluded due to illness, and another 146 were excluded because they either provided incomplete data or because the accelerometer malfunctioned. Another 380 participants were excluded from the study because they did not meet the specific criteria for accelerometer wear time. Hence, the final sample comprised 336 participants. Detailed characteristics of the study sample are summarized in Table 1.

**Table 1:** Descriptive characteristics of the study sample

	Boys (*n* = 128)		Girls (*n* = 208)		*p* value^c^
	Mean^a^	var^b^	Mean^a^	var^b^	
Age (years)	14.4	2.4	13.8	2.6	0.033
Height (cm)	167.0	14.4	158.3	11.9	< 0.001
Weight (kg)	58.6	17.5	51.4	13.1	< 0.001
Fat mass (%)	15.5	7.6	23.7	8.0	< 0.001
Fat mass index (kg/m^2^)	3.4	2.4	5.0	2.5	< 0.001
**Weight status** (% of *n*)^d^					
Underweight	2.3		1.0		0.341
Normal weight	74.2		76.4		0.649
Overweight	14.8		18.3		0.407
Obese	8.7		4.3		0.099
**24-h movement behaviors** (min/day)^d^					
Sleep (min/day)	479.1	15.0	486.3	13.7	0.989
SB school (min/day)	229.4	19.1	215.2	19.6	0.236
SB out-of-school (min/day)	427.5	17.9	394.9	17.1	0.056
LPA school (min/day)	89.4	13.9	90.5	13.6	0.634
LPA out-of-school (min/day)	142.0	14.7	159.9	13.9	0.011
MPA school (min/day)	23.5	13.9	26.3	15.8	0.005
MPA out-of-school (min/day)	44.4	13.5	62.4	13.7	< 0.001
VPA school (min/day)	2.4	28.8	1.6	34.7	< 0.001
VPA out-of-school (min/day)	2.3	63.2	2.9	57.9	0.153

*LPA* light physical activity, *MPA* moderate physical activity, *SB* sedentary behaviors, *VPA* vigorous physical activity

^a^Geometric mean for time-use components; arithmetic mean for non-compositional continuous variables; percentages for categorical variables

^b^The part of total variance related to a given time-use component; standard deviation for other variables

^c^Differences between sexes were tested using the *t*-test for independent samples and the chi-squared test for categorical variables

^d^Based on the BMI *z*-score

### Device-measured 24-h movement behaviors

SB, PA, and sleep were measured using the wrist-worn ActiGraph accelerometer GT9X Link or wGT3X-BT (ActiGraph Corp., Pensacola, FL, USA) which are comparable if count-based metrics are used [23]. Participants were given an accelerometer at their schools and asked to wear it for seven consecutive days, except when bathing and swimming. The devices were initialized to collect data at 100 Hz, and data were downloaded using the ActiLife software version 6.13.3 (ActiGraph Corp., Pensacola, FL, USA).

Raw accelerometer data were saved as GT3X files and reintegrated to 5-s periods. Non-wear time was captured with the Choi algorithm [24] to minimize misclassification of wear time as non-wear time during the sleep time period [25] and removed for further analysis. Vector magnitude activity counts per 5 s were used to estimate SB (< 306 counts), light physical activity (LPA; 306–817 counts), moderate physical activity (MPA; 818–1968 counts), and vigorous physical activity (VPA; ≥ 1969 counts) [26]. Participants’ log diaries were used to specify school (time between the start and the end of school) and out-of-school movement behaviors (all remaining time). Sleep duration was derived using the Cole-Kripke algorithm [27] which provides high accuracy of sleep estimation against polysomnography in the pediatric population [28].

Time spent in SB, LPA, MPA, VPA, and sleep were summed and then normalized to 24 h (i.e., 1440 min). We applied the wear time requirement of ≥ 1200 min of a total wear time with ≥ 720 min of awake time, ≥ 320 min of sleep time, and 220–440 min of school time to constitute a valid measurement. In order to cover the whole period of 1 week, participants with a valid set of 7 days and nights and with valid school and out-of-school segments for all the five school days included in the measurement period were included in the final analysis.

### Adiposity indicators

Adiposity was assessed on the school premises during school hours using the multi-frequency bioelectrical impedance (InBody 720, InBody Co., Seoul, South Korea; 1–1000 kHz). Fat mass percentage (FM%) and fat mass index (FMI) were used as indicators of adiposity. The multi-frequency bioimpedance analysis method has been previously validated against dual-energy X-ray absorptiometry for assessing adiposity in pediatric population [29]. Participants were instructed in advance to avoid any vigorous exercise for at least a day before the examination and to maintain proper hydration.

### Covariates

The several covariates were selected to control for potential cofounding factors. We selected a set of potential confounding factors a priori based on the literature [30] and based on the preliminary analysis. Data on participants’ active transport from school (defined as any mode of non-motorized transportation on most days of the school week) and unhealthy diet (defined as a low frequency in consumption of fruits and vegetable) were self-reported. Maternal overweight and obesity (self-reported body mass index ≥ 25 kg/m^2^) and education level were reported by parents. Missing values (2–6%) were imputed using the *k*-nearest neighbor imputation algorithm [31].

### Statistical analyses

IBM SPSS Statistics version 23 (IBM, Armonk, NY, USA) was used to determine the descriptive analyses and to verify the data for further analysis. A CoDA was conducted with R Statistical Software, version 4.0.2 (R Foundation for Statistical Computing, Vienna, Austria) using the robCompositions package. The analysis was performed separately for boys and girls to take into account sexual dimorphism in adiposity [30].

The descriptive statistics of the compositional variables are presented as a robust center (i.e., vector of compositional means) and variation matrix (i.e., dispersion of compositional data). We used the 9-part 24-h movement behavior composition which takes into account school and out-of-school behaviors (i.e., SB, LPA, MPA, and VPA) and sleep. The variables of movement behaviors were expressed as isometric log-ratio coordinates and the first pivot coordinate (*ilr*_1_), which includes all relative information about the one part given the remaining parts of 24-h composition, was used in the analysis [32]. The order of movement behavior variables was rearranged in every given analysis to be able to analyze each part of the 24-h composition using the first pivot coordinate system [20]. A few zero values in school VPA (seven cases) were replaced using the Bayesian-multiplicative method [33].

Differences between sexes were tested using the independent *t*-test for scale variables and with the chi-square test for categorical variables. The compositional regression analysis adjusted for covariates, age, region, and season of data collection were used to examine associations between components of the 24-h movement behaviors and adiposity. The robust estimators were used to suppress the influence of outlying observations [34] that occurred in school and out-of-school VPA. Dependent variables were transformed using a natural logarithm before analysis to achieve the absolute scale and a normal distribution. The sample size of both sex categories was sufficient to detect at least a medium effect size (*f*^2^ ≤ 0.15) in the population and to ensure a statistical power of ≥ 80% and an alpha error of 0.05 for regression models with 13 explanatory variables [35].

The compositional isotemporal substitution model [21] was used to estimate relative differences in adiposity indicators associated with one-to-one reallocations from time spent in SB to PA of different intensities within school and out-of-school time. Estimated differences in adiposity indicators were considered significant when 95% confidence intervals (CIs) did not cover zero.

## Results

The descriptive characteristics of the included participants did not differ from those who were excluded (Table S1). The characteristics of the study participants included in the final sample are shown in Table 1. Boys had significantly lower FM% and FMI, by 8.2% points and 1.6 kg/m^2^ (*p* < 0.001 for both), respectively, compared with girls. In both age categories, participants spent 64–69% of their school and out-of-school time in SB. Relative to the remaining 24-h movement behaviors, boys spent less time in school MPA, by 2.8 min/day (*p* = 0.005), and more time in school VPA, by 0.8 min/day (*p* < 0.001), than girls. Boys also spent less time in out-of-school LPA and out-of-school MPA, by 17.9 min/day (*p* = 0.011) and 18 min/day (*p* < 0.001), respectively.

Table 2 displays the results of the compositional regression analysis between all 24-h movement behaviors and adiposity indicators. Significant associations were found between out-of-school SB (relative to the remaining 24-h movement behaviors) and FM% for both boys (*β*_ilr1_ = 0.63, 95% CI = 0.03 to 1.22) and girls (*β*_ilr1_ = 0.62, 95% CI = 0.25 to 0.98). Similar associations were found for FMI for boys (*β*_ilr1_ = 0.76, 95% CI = 0.03 to 1.49) and for girls (*β*_ilr1_ = 0.80, 95% CI = 0.28 to 1.32). Relative to the remaining 24-h movement behaviors, out-of-school LPA was significantly associated with FM% (*β*_ilr1_ = − 0.43, 95% CI = −0.86 to −0.01) in girls. No significant associations between movement behaviors during school and adiposity indicators were found.

**Table 2:** Robust compositional regression analysis of associations between 24-h movement behaviors and adiposity indicators

	Boys (*n* = 128)						Girls (*n* = 208)					
	Fat mass^a^ (%)			Fat mass index^a^ (kg/m^2^)			Fat mass^a^ (%)			Fat mass index^a^ (kg/m^2^)		
	*β*_ilr1_^b^	(95% CI)	*p* value	*β*_ilr1_^b^	(95% CI)	*p* value	*β*_ilr1_^b^	(95% CI)	*p* value	*β*_ilr1_^b^	(95% CI)	*p* value
Sleep (min/day)	−0.67	(−1.62, 0.27)	0.159	−0.65	(−1.92, 0.63)	0.318	−0.46	(−0.96, 0.05)	0.075	−0.71	(−1.45, 0.03)	0.058
SB school (min/day)	−0.32	(− 1.09, 0.45)	0.411	−0.51	(−1.49, 0.46)	0.297	−0.20	(−0.58, 0.18)	0.302	−0.20	(−0.76, 0.35)	0.473
SB out-of-school (min/day)	0.63	(0.03, 1.22)	0.040	0.76	(0.03, 1.49)	0.040	0.62	(0.25, 0.98)	0.001	0.80	(0.28, 1.32)	0.003
LPA school (min/day)	0.18	(−0.54, 0.90)	0.615	0.17	(−0.68, 1.02)	0.691	0.38	(−0.06, 0.81)	0.086	0.52	(−0.09, 1.12)	0.094
LPA out-of-school (min/day)	0.11	(−0.50, 0.71)	0.732	0.20	(−0.50, 0.90)	0.569	−0.43	(−0.86, − 0.01)	0.047	−0.56	(−1.21, 0.08)	0.086
MPA school (min/day)	0.23	(−0.19, 0.65)	0.284	0.30	(−0.19, 0.78)	0.230	−0.08	(−0.41, 0.25)	0.636	−0.07	(−0.54, 0.39)	0.765
MPA out-of-school (min/day)	−0.02	(−0.56, 0.52)	0.940	−0.14	(−0.73, 0.45)	0.640	0.20	(−0.19, 0.59)	0.305	0.25	(−0.33, 0.83)	0.388
VPA school (min/day)	−0.05	(−0.16, 0.05)	0.298	−0.07	(−0.19, 0.06)	0.281	0.04	(−0.02, 0.09)	0.200	0.05	(−0.03, 0.13)	0.219
VPA out-of-school (min/day)	−0.08	(−0.21, 0.06)	0.254	−0.06	(−0.22, 0.09)	0.430	−0.07	(−0.15, 0.01)	0.077	−0.07	(−0.18, 0.04)	0.190

*CI* confidence interval, *ilr1* isometric log-ratio (first coordinate), *LPA* light physical activity, *MPA* moderate physical activity, *SB* sedentary behaviors, *VPA* vigorous physical activity

^a^Variable was transformed using natural logarithm before analysis

^b^Regression coefficient for fully-adjusted model

Independent variables are expressed as the first pivot coordinate which represents the relative contribution of one behavior relative to remaining behaviors within a 24-hour cycle. Results were derived from nine regression models corresponding to the pivot coordinate representations of specific time-use components

**Fig. 1:**
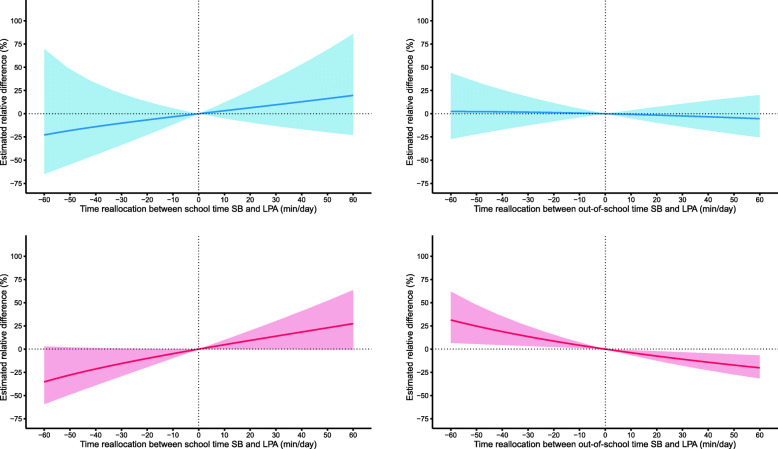
Relative difference in fat mass percentage associated with replacing school (left) and out-of-school (right) SB with LPA in boys (top) and girls (bottom). Positive values on *x*-axis present time replacement from context-specific SB to LPA, and negative values time replacement from context-specific LPA to SB

Estimated relative differences in adiposity indicators associated with simulated reallocations of time from school and out-of-school SB to PA of different intensities are presented in Tables 3 and 4. In girls, substituting 30 min/day of out-of-school SB to out-of-school LPA was associated with lower FM% and FMI by 10.1% (95% CI = 3.3 to 17.9) and 14% (95% CI = 2.7 to 24.0), respectively. Estimated differences in adiposity were not exactly symmetrical because higher FM% by 13.5% (95% CI = 3.3 to 24.7) and FMI by 18% (95% CI = 2.3 to 36.1) were associated with a 30-min reallocation from LPA to SB within out-of-school time. No significant change in adiposity indicators was found for boys (Fig. 1) and for time reallocations between context-specific SB and MPA and VPA (Figures S1 and S2).

**Table 3:** Estimated relative difference in adiposity associated with replacing context-specific sedentary behaviors with physical activity among boys (*n* = 128)

	LPA 30 min/day reallocation		MPA 10 min/day reallocation		VPA 2 min/day reallocation	
	Percentage change	(95% CI)	Percentage change	(95% CI)	Percentage change	(95% CI)
**Fat mass (%)**						
SB to PA (school time)	9.6	(−13.3, 38.6)	9.4	(−4.5, 25.4)	−2.8	(−8.6, 3.4)
PA to SB (school time)	−10.1	(−33.6, 21.6)	−12.4	(−29.3, 8.5)	9.9	(−8.8, 32.3)
SB to PA (out-of-school time)	−2.4	(−14.0, 10.8)	−1.8	(−11.1, 8.5)	−4.6	(−11.8, 3.1)
PA to SB (out-of-school time)	1.7	(−12.5, 18.2)	1.9	(−10.1, 15.5)	15.4	(−9.5, 47.1)
**Fat mas index (kg/m**^**2**^**)**						
SB to PA (school time)	12.2	(−15.0, 48.1)	12.8	(−3.7, 32.1)	−3.5	(−10.4, 4.1)
PA to SB (school time)	−11.8	(−38.3, 26.1)	−16.1	(−34.4, 7.5)	12.5	(−10.2, 41.0)
SB to PA (out-of-school time)	−1.6	(−14.9, 13.8)	−4.3	(−14.1, 6.7)	−3.9	(−12.5, 5.5)
PA to SB (out-of-school time)	0.4	(−15.5, 19.2)	5.1	(−8.3, 20.5)	12.7	(−15.8, 50.7)

*CI* confidence interval, *LPA* light physical activity, *MPA* moderate physical activity, *PA* physical activity, *SB* sedentary behaviors, *VPA* vigorous physical activity

**Table 4:** Estimated relative difference in adiposity associated with replacing context-specific sedentary behaviors with physical activity among girls (*n* = 208)

	LPA 30 min/day reallocation		MPA 10 min/day reallocation		VPA 2 min/day reallocation	
	Percentage change	(95% CI)	Percentage change	(95% CI)	Percentage change	(95% CI)
**Fat mass (%)**						
SB to PA (school time)	13.9	(−0.4, 30.3)	− 1.5	(−10.9, 8.9)	3.8	(−1.4, 7.7)
PA to SB (school time)	−15.5	(−29.1, 0.8)	2.8	(−11.4, 19.2)	−9.7^a^	(−22.7, 5.4)
SB to PA (out-of-school time)	−10.1	(−17.9, −3.3)	1.4	(−4.1, 7.1)	−3.7	(−7.3, 0.1)
PA to SB (out-of-school time)	13.5	(3.3, 24.7)	−1.9	(−8.0, 4.7)	8.5	(−0.5, 18.3)
**Fat mas index (kg/m**^**2**^**)**						
SB to PA (school time)	18.3	(−1.5, 42.2)	−1.2	(−14.2, 13.7)	4.1	(−2.1, 10.7)
PA to SB (school time)	−19.9	(−37.1, 2.1)	2.3	(−16.9, 25.9)	−12.8^a^	(−29.7, 8.3)
SB to PA (out-of-school time)	−14.0	(−24.0, −2.7)	1.6	(−6.3, 10.2)	−4.0	(−9.1, 1.4)
PA to SB (out-of-school time)	18.0	(2.3, 36.1)	−2.3	(−11.1, 7.5)	9.0	(−3.7, 23.4)

*CI* confidence interval, *LPA* light physical activity, *MPA* moderate physical activity, *PA* physical activity, *SB* sedentary behaviors, *VPA* vigorous physical activity

^a^Estimate is based on 1.5 min/day reallocation because the model was unable to estimate differences due to the low compositional mean of school VPA

## Discussion

The findings of the present study provide novel insights into the compositional associations of context-specific SB with adiposity and the changes in adiposity associated with replacing SB with PA. We found that boys and girls spent approximately two thirds of their school and out-of-school time being sedentary. The greater amount of time spent in out-of-school SB was associated with FM% and FMI in both sexes. Replacing out-of-school SB with out-of-school LPA results in favorable changes in adiposity among girls. No significant associations were found for school SB.

In line with previous studies [11–14, 36, 37], our participants spent a predominant proportion of school and out-of-school time being sedentary. Thus, these context-specific settings present an opportunity for interventions targeting obesity prevention by replacing SB with PA [38, 39]. Our analysis, however, did not reveal any significant differences in adiposity associated with replacing school SB with school PA of different intensities. A plausible explanation behind this finding may be that school movement behaviors have low intrapersonal variability [12] and may not differ between individuals with normal and excess adiposity. Another explanation is that we did not take into account patterns of SB. A recent study found significant compositional associations between time spent in middle sedentary bouts (i.e., bouts of 10–29 min) and adiposity in children of a school-going age, whilst no associations were found for total SB [40]. SB patterns should therefore be considered when examining associations between SB and adiposity.

We had no empirical data on SB patterns during school time, so we could only hypothesize that most of the time our participants spent in school SB was accumulated by a few periods of prolonged SB (i.e., during lessons). Prolonged uninterrupted SB is associated with several adverse health outcomes including excess adiposity [40–42]. Consequently, breaking up prolonged sitting with short bouts of PA could make a significant contribution to obesity prevention. This assumption is supported by a recent CoDA-based study in which favorable adiposity status was associated with time reallocation from middle sedentary bouts to moderate-to-vigorous PA [40]. Moreover, clinical trials also reporting that incorporating brief bouts of PA into school time may attenuate increases in adiposity [43].

Out-of-school time also presents numerous opportunities to prevent excess adiposity by reducing SB in favor of PA. Although a plethora of studies show that a favorable adiposity status is associated with at least MPA as part of active transportation to and from school [44] and organized sport and PA [45], the present study did not find such associations for both out-of-school MPA and VPA. These different results could be due to the fact that the independent variables used in the present study included relative information about all the parts of 24-h movement behaviors composition (i.e., the first pivot coordinate), whilst previously published studies were not based on 24-h movement behavior data and/or had not used adequate statistical methods for time-use data. For example, omitting sleep from the analysis may yield biased estimates of the association of out-of-school PA with adiposity. Sleep represents a predominant part of 24-h movement behavior composition and has been found to be independently associated with childhood obesity [46]. Moreover, inadequate sleep is associated with less healthy awake time, especially with higher levels of prolonged SB [18]. Future studies should consider a 24-h time-use paradigm and use appropriate statistical methods.

The novel finding of this study is that adiposity could be improved by increasing out-of-school LPA at the expense of time spent in out-of-school SB. This finding corresponds with previous CoDA-based studies showing the favorable difference in adiposity [47] and body mass index *z*-score [11] for time reallocations between total SB and LPA. Moreover, the recent study showed that LPA moderates associations between sitting and adiposity indicators among adolescents [48]. Although LPA requires a low consumption of energy, it has the potential of improving health outcomes. The main reason for this is that LPA represents the dominant part of out-of-school time composition and includes various mixes of daily living activities, which may interrupt prolonged SB. The experimental study by Bailey and Locke [49] supports this assumption by showing that interrupting SB with frequent sporadic bouts of LPA leads to enhanced cardiometabolic health in the adult population. However, the potential of LPA for the prevention of excess adiposity in the pediatric population is largely unexplored and undocumented. Future studies are warranted to gain a better understanding of the role of LPA in the prevention of childhood obesity.

The compositional isotemporal substitution model used in the present study found significant differences in adiposity only in girls. Similar to our findings, previously published studies [47, 50] confirmed sex-specific differences in adiposity associated with time reallocations between SB and LPA. This finding can be partly explained by differences in out-of-school activity. In the present study, boys spent significantly less time in out-of-school LPA and out-of-school MPA than girls. Additionally, differences in overweight and obesity prevalence between sex categories indicate differences in variability of adiposity between boys and girls, which may also have caused the sex-specific results of the analysis.

To the best of our knowledge, this is the first study to use CoDA to examine associations between context-specific SB and adiposity indicators. The strength of the study is the availability of raw multi-day 24-h movement data which allowed SB adjustment for PA and sleep duration. There are also some limitations to this study. First, the design of study was cross-sectional with limited ability to draw causality in the associations between 24-h movement behaviors and adiposity. Second, an isotemporal substitution model provides only theoretical estimates of changes in adiposity. Our findings should be, therefore, interpreted with caution and need to be contrasted in experimental designs. Third, although the regression analysis was adjusted for several confounders, there are still several potential endogenous and exogenous factors that have not been considered. Last, although the sample size is comparable with previous studies [11, 50, 51], it allowed us to detect at least a medium effect size in the population.

## Conclusion

The present study is the first to use CoDA when examining associations between context-specific SB and adiposity status in school-aged children and adolescents. We revealed positive (unfavorable) associations between out-of-school SB and adiposity. Based on compositional isotemporal substituting modeling, the present study suggests that adiposity status could be improved by replacing out-of-school SB with out-of-school LPA. No significant associations were found for school SB. This study, therefore, highlights the need for assessing the context-specific SB and to amplify the benefits of LPA in the prevention of excess adiposity. Findings from the present study provide further evidence for public health policy and practices and may help to design more effective strategies to prevent childhood obesity.

## Supplementary Information

**Additional file 1: Figure S1.** Relative difference in fat mass percentage associated with replacing school (left) and out-of-school (right) SB with MPA in boys (top) and girls (bottom). Positive values on the x-axis present time replacement from context-specific SB to MPA, and negative values time replacement from context-specific MPA to SB.

**Additional file 2: Figure S2.** Relative difference in fat mass percentage associated with replacing school (left) and out-of-school (right) SB with VPA in boys (top) and girls (bottom). Positive values on x-axis present time replacement from context-specific SB to VPA, and negative values time replacement from context-specific VPA to SB. *Note*: The isotemporal substitution model was unable to estimate difference for reallocation between school SB and VPA due to low compositional mean for school VPA among girls.

**Additional file 3: Table S1.** Descriptive characteristics of included and excluded participants.

## Data Availability

The dataset analyzed during the current study are available from the corresponding author on reasonable request.
