# Supplementary figures and images for: Replacing school and out-of-school sedentary behaviors with physical activity and its associations with adiposity in children and adolescents: a compositional isotemporal substitution analysis
Source: Environ Health Prev Med. 2021 Jan 27;26:16. doi: 10.1186/s12199-021-00932-6 (PMC7842010; doi:10.1186/s12199-021-00932-6)

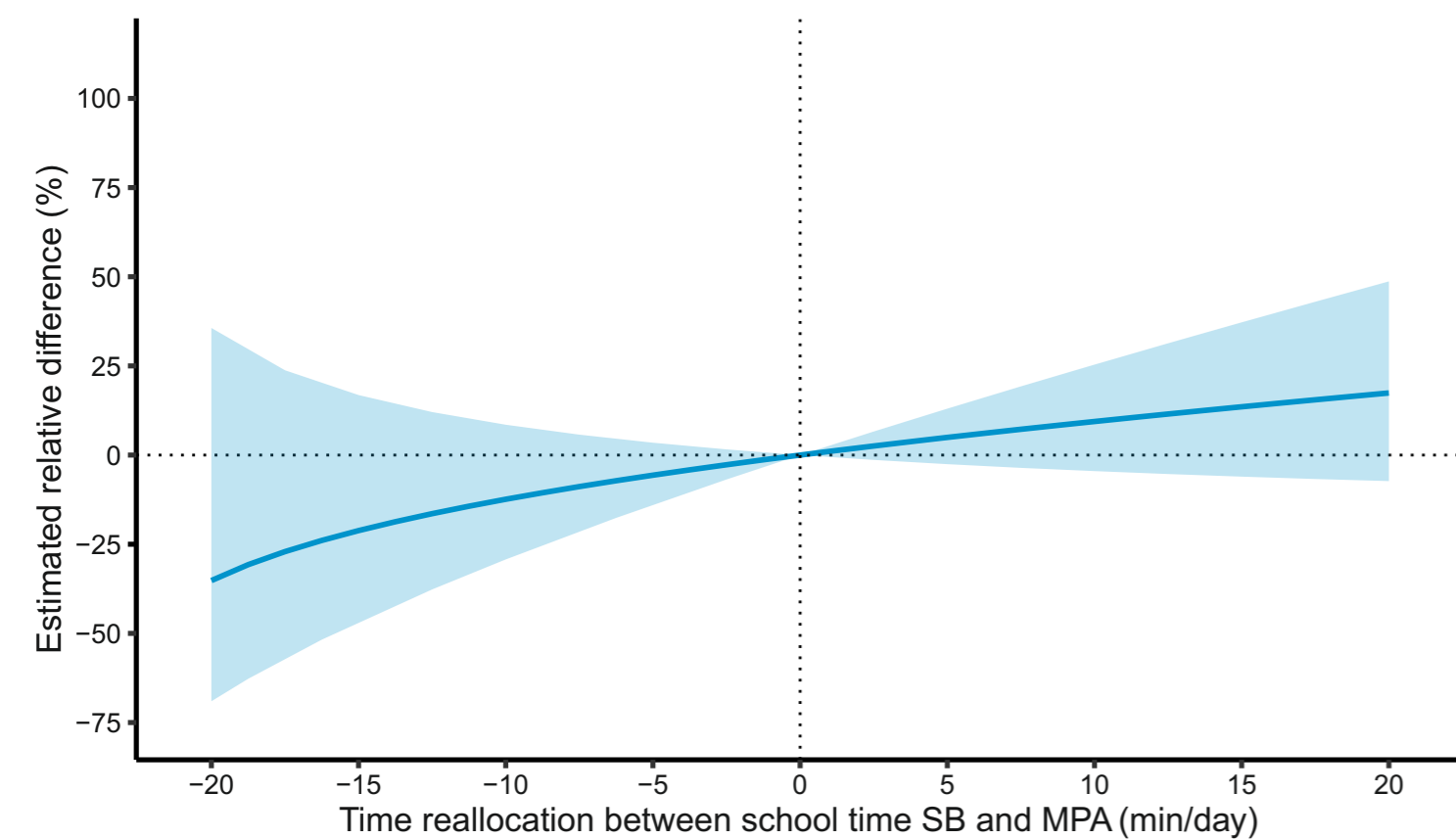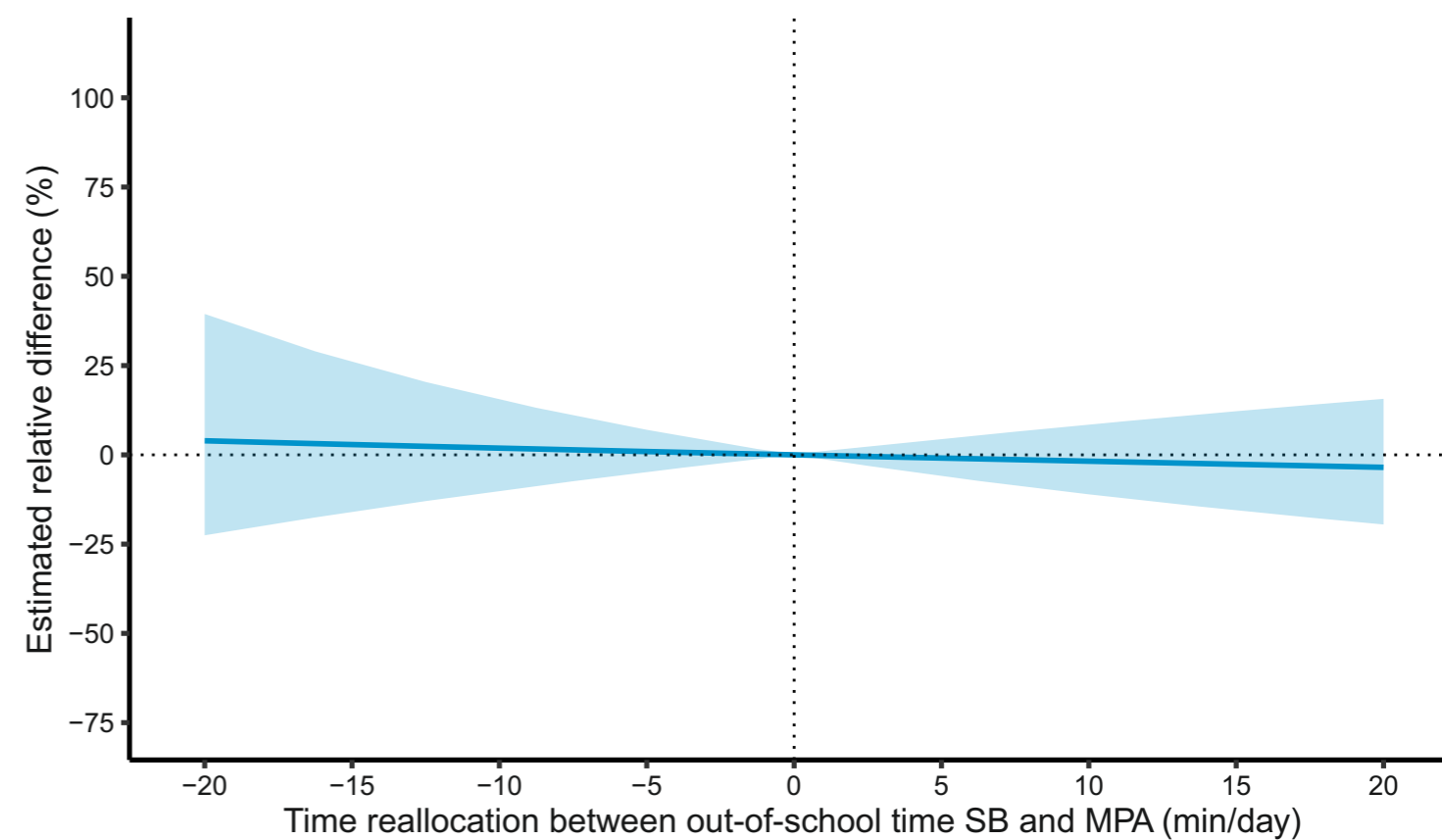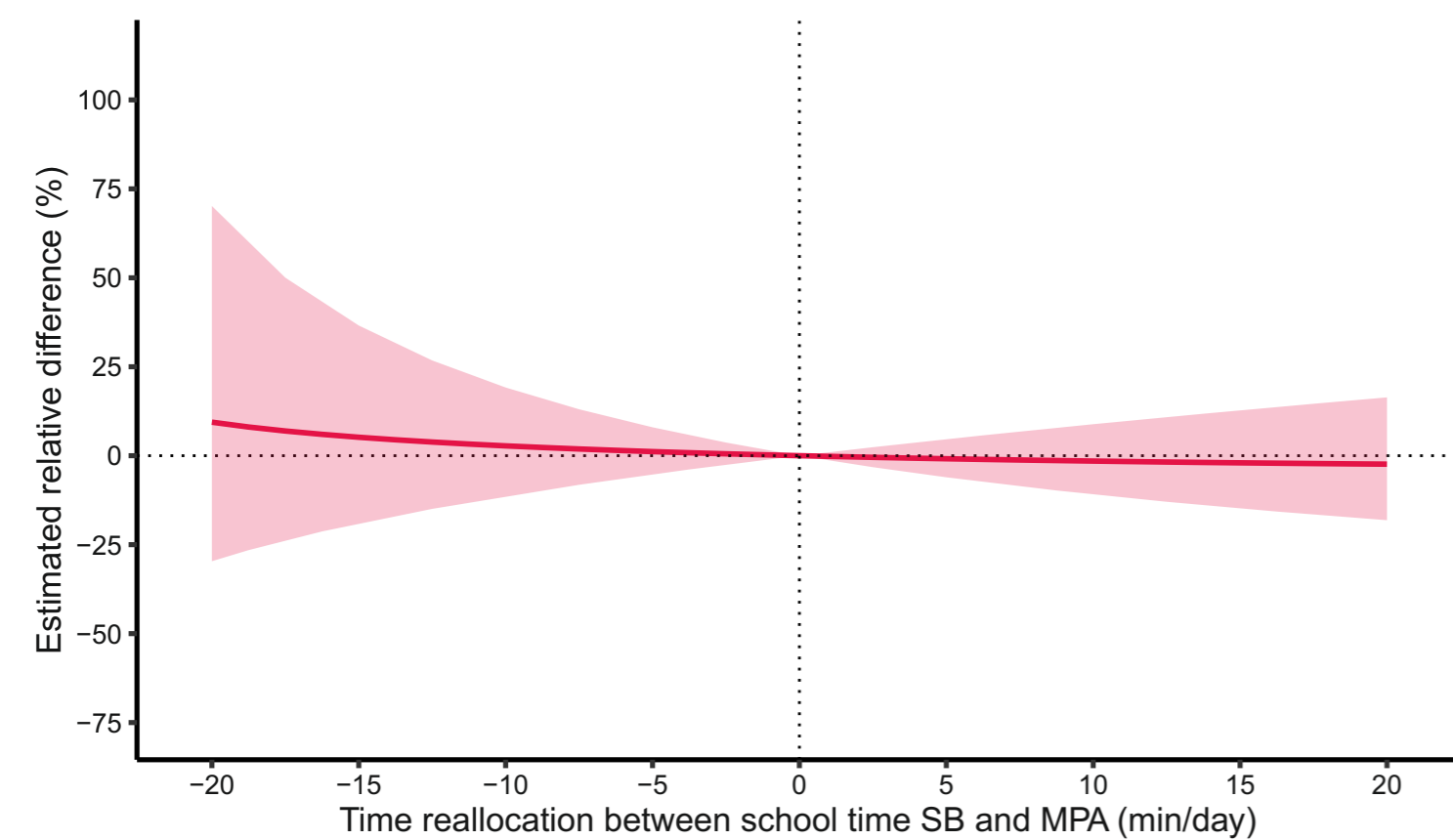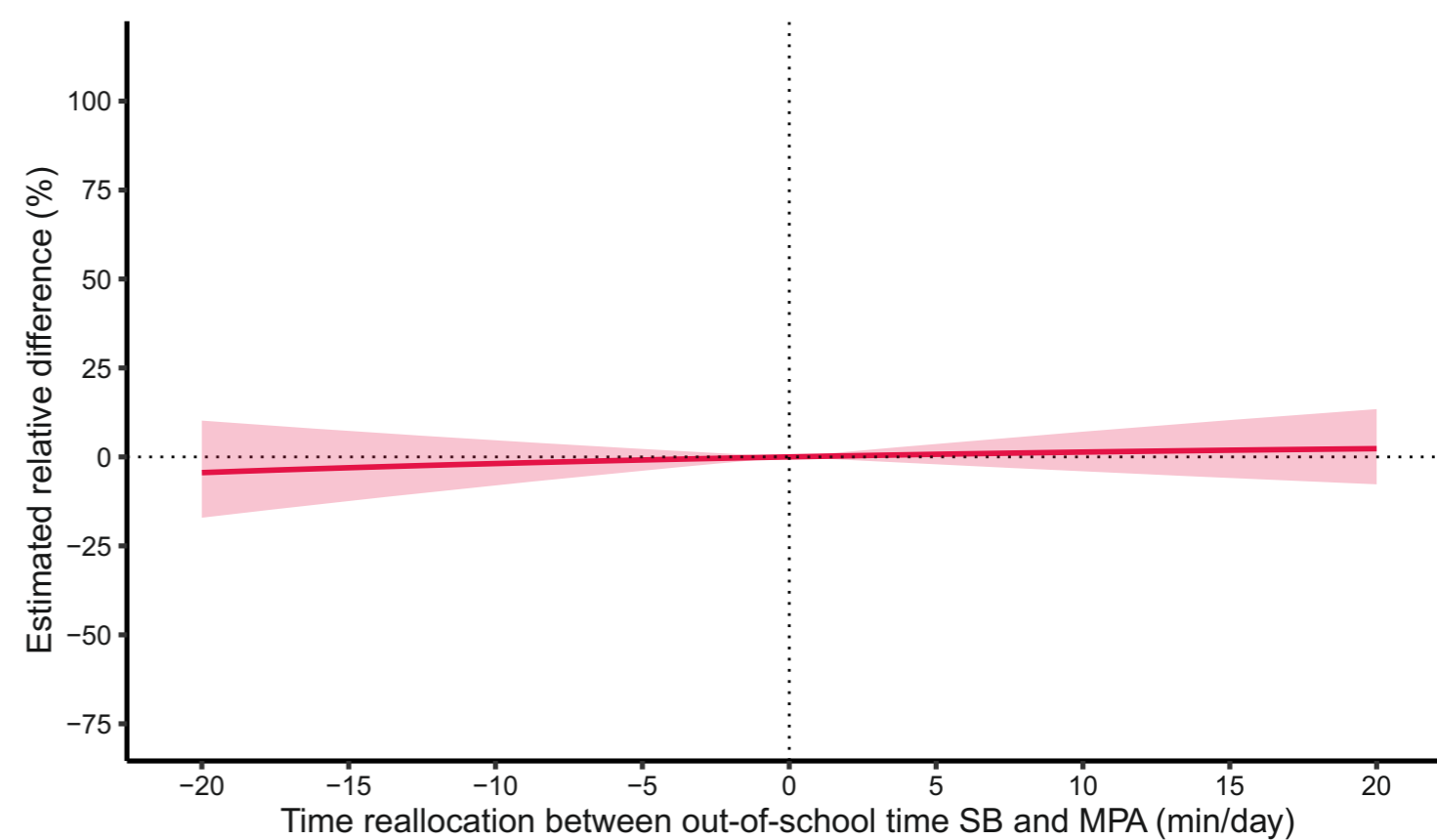

Supplement: Supplementary file 1 — Additional file 1: Figure S1. Relative difference in fat mass percentage associated with replacing school (left) and out-of-school (right) SB with MPA in boys (top) and girls (bottom). Positive values on the x-axis present time replacement from context-specific SB to MPA, and negative values time replacement from context-specific MPA to SB. [file 12199_2021_932_MOESM1_ESM.pdf]

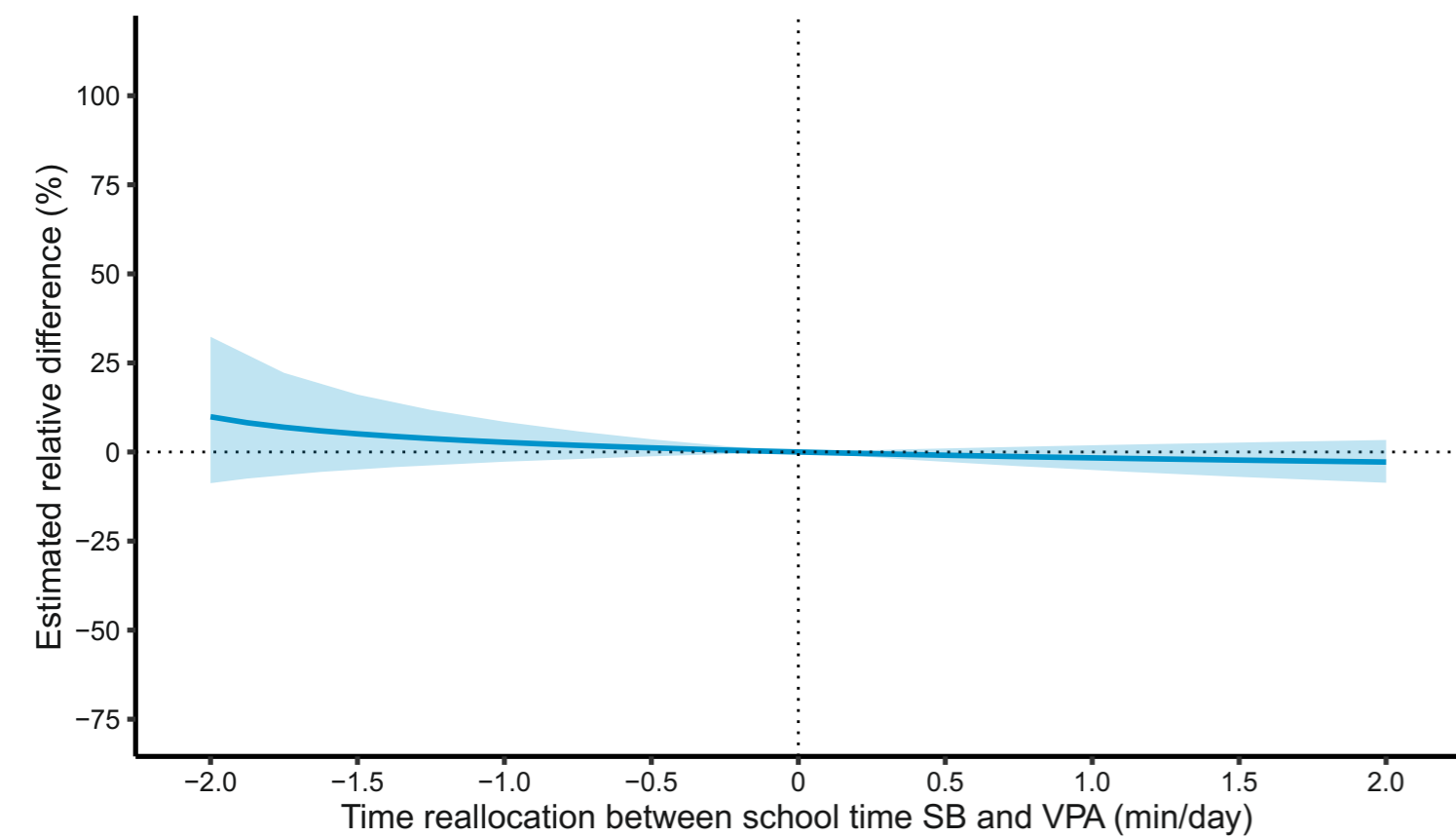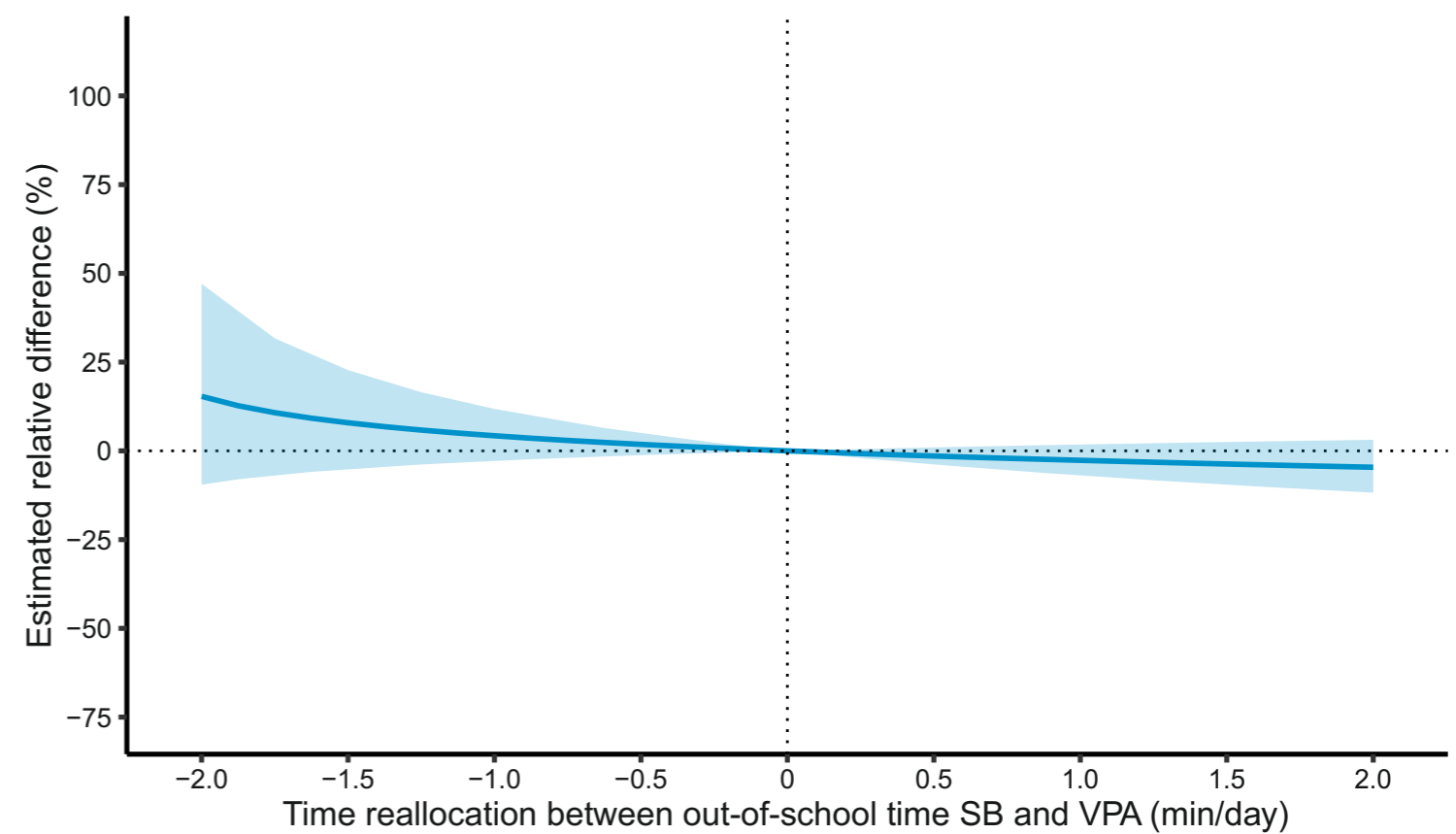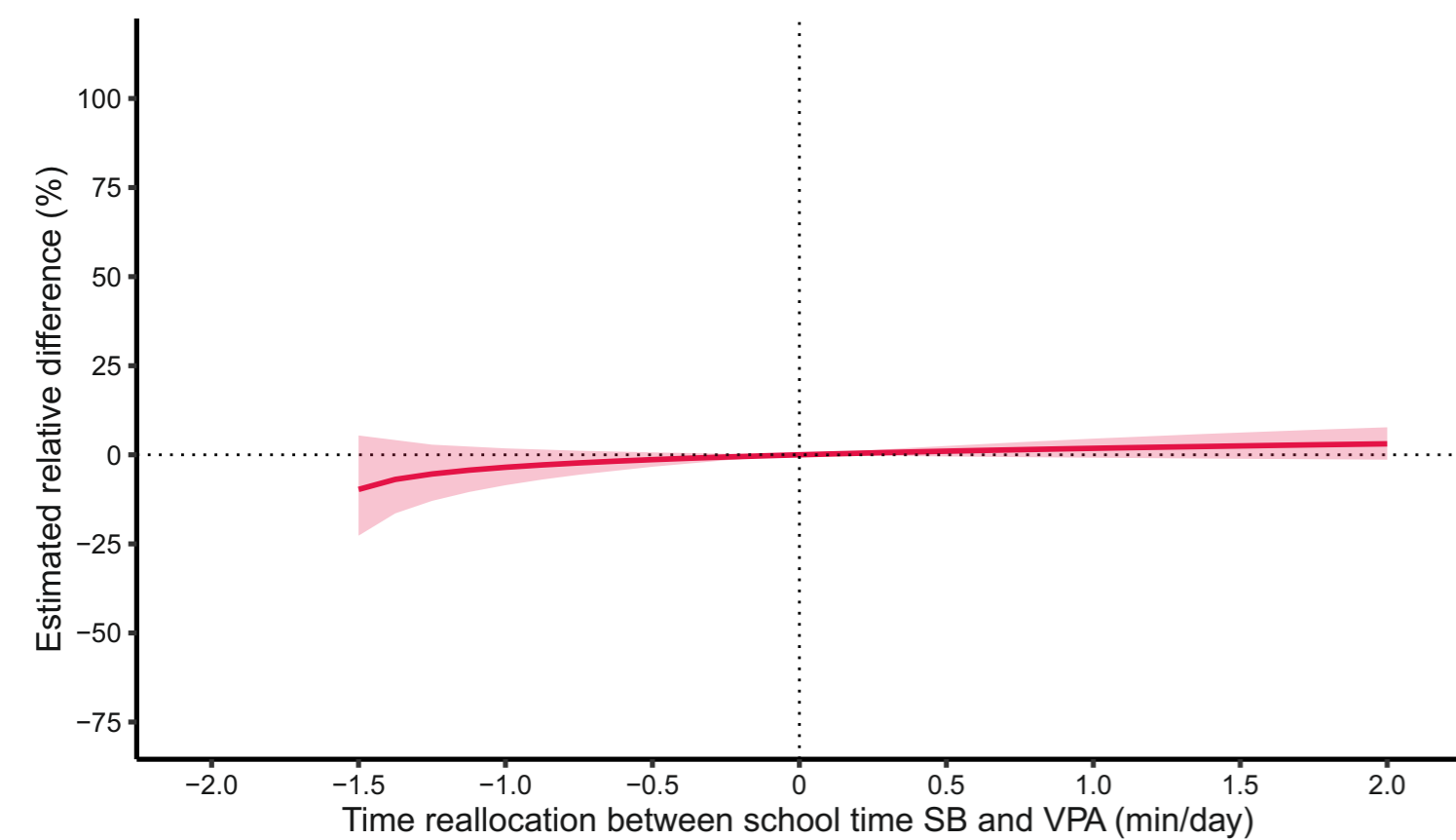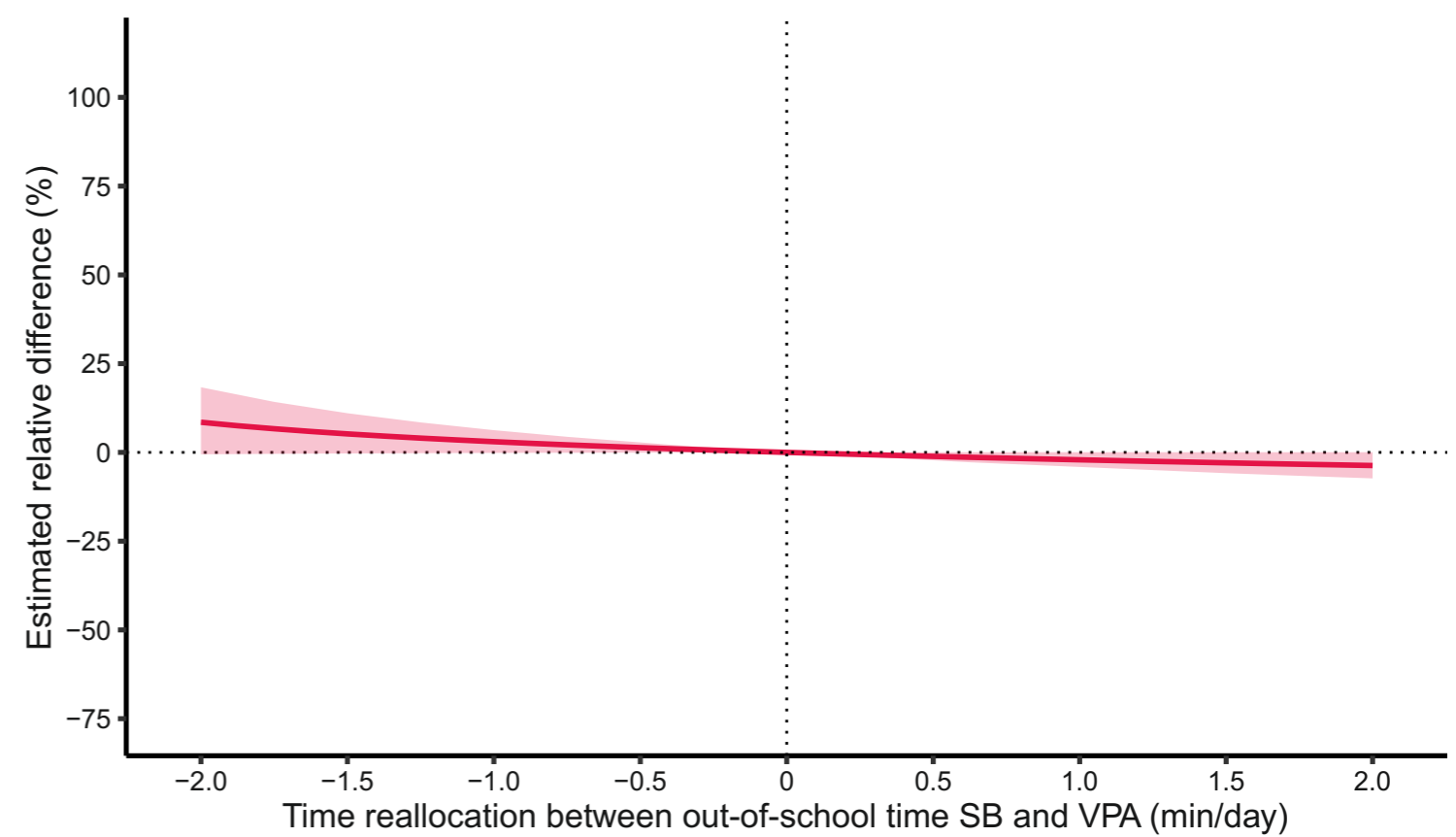

Supplement: Supplementary file 2 — Additional file 2: Figure S2. Relative difference in fat mass percentage associated with replacing school (left) and out-of-school (right) SB with VPA in boys (top) and girls (bottom). Positive values on x-axis present time replacement from context-specific SB to VPA, and negative values time replacement from context-specific VPA to SB. Note: The isotemporal substitution model was unable to estimate difference for reallocation between school SB and VPA due to low compositional mean for school VPA among girls. [file 12199_2021_932_MOESM2_ESM.pdf]
