# Supplementary material for: Replacing school and out-of-school sedentary behaviors with physical activity and its associations with adiposity in children and adolescents: a compositional isotemporal substitution analysis
Source: Environ Health Prev Med. 2021 Jan 27;26:16. doi: 10.1186/s12199-021-00932-6 (PMC7842010; doi:10.1186/s12199-021-00932-6)
Supplement: Supplementary file 3 — Additional file 3: Table S1. Descriptive characteristics of included and excluded participants. [file 12199_2021_932_MOESM3_ESM.docx]

### Table S1. Descriptive characteristics of included and excluded participants

|  |  |  | Included  (*n* = 336) | |  | Excluded  (*n* = 571) | |  | *p*-value^a^ |
| --- | --- | --- | --- | --- | --- | --- | --- | --- | --- |
|  |  |  | Mean | SD |  | Mean | SD |  |  |
| Age (years) |  |  | 14.1 | 2.5 |  | 19.9 | 2.6 |  | 0.342 |
| Height (cm) |  |  | 161.6 | 13.6 |  | 161.2 | 14.4 |  | 0.652 |
| Weight (kg) |  |  | 54.2 | 15.3 |  | 53.7 | 15.5 |  | 0.644 |
| Fat mas (%) |  |  | 20.6 | 8.8 |  | 19.4 | 8.9 |  | 0.067 |
| Fat mass index (kg/m^2^) |  |  | 4.4 | 2.6 |  | 4.2 | 2.8 |  | 0.202 |
| SD, standard deviation.  ^a^ Differences between sexes were tested using the *t*-test for independent samples. | | | | | | | | | |
